# Preparation and characterization of bovine dental pulp-derived extracellular matrix hydrogel for regenerative endodontic applications: an in vitro study

**DOI:** 10.1186/s12903-024-05004-z

**Published:** 2024-10-24

**Authors:** Hisham Elnawam, Abdelrahman Thabet, Ahmed Mobarak, Amr Abdallah, Rania Elbackly

**Affiliations:** 1 Endodontics, Conservative Dentistry Department, Faculty of Dentistry, Alexandria University, Alexandria, Egypt; 2 Tissue Engineering Laboratories, Faculty of Dentistry, Alexandria University, Alexandria, Egypt

**Keywords:** Biomimetic scaffolds, Dental pulp-derived extracellular matrix, Dentin-pulp regeneration, Extracellular matrix hydrogels, Hyaluronic acid, Regenerative endodontics

## Abstract

**Background:**

The use of biological scaffolds in regenerative endodontics has gained much attention in recent years. The search for a new biomimetic scaffold that contains tissue-specific cell homing factors could lead to more predictable tissue regeneration. The aim of this study was to prepare and characterize decellularized bovine dental pulp-derived extracellular matrix (P-ECM) hydrogels for regenerative endodontic applications.

**Methods:**

Freshly extracted bovine molar teeth were collected. Bovine dental pulp tissues were harvested, and stored at -40º C. For decellularization, a 5-day protocol was implemented incorporating trypsin/EDTA, deionized water and DNase treatment. Decellularization was evaluated by DNA quantification and histological examination to assess collagen and glycosaminoglycans (GAGs) content. This was followed by the preparation of P-ECM hydrogel alone or combined with hyaluronic acid gel (P-ECM + HA). The fabricated scaffolds were then characterized using protein quantification, hydrogel topology and porosity, biodegradability, and growth factor content using Enzyme-linked immunosorbent assay (ELISA): transforming growth factor beta-1(TGF-β1), basic fibroblast growth factor (bFGF), bone morphogenetic protein 2 (BMP-2) and vascular endothelial growth factor (VEGF).

**Results:**

Decellularization was histologically confirmed, and DNA content was below (50 ng/mg tissue). P-ECM hydrogel was prepared with a final ECM concentration of 3.00 mg/ml while P-ECM + HA hydrogel was prepared with a final ECM concentration of 1.5 mg/ml. Total protein content in P-ECM hydrogel was found to be (439.0 ± 123.4 µg/µl). P-ECM + HA showed sustained protein release while the P-ECM group showed gradual decreasing release. Degradation was higher in P-ECM + HA which had a significantly larger fiber diameter, while P-ECM had a larger pore area percentage. ELISA confirmed the retention and release of growth factors where P-ECM hydrogel had higher BMP-2 release, while P-ECM + HA had higher release of TGF-β1, bFGF, and VEGF.

**Conclusions:**

Both P-ECM and P-ECM + HA retained their bioactive properties demonstrating a potential role as functionalized scaffolds for regenerative endodontic procedures.

**Supplementary Information:**

The online version contains supplementary material available at 10.1186/s12903-024-05004-z.

## Background

Regenerative endodontic Procedures (REPs) are considered a paradigm shift in clinical endodontics. REPs employ stem cells, scaffolds, and bioactive growth factors as a tissue engineering triad to regenerate the dentin-pulp complex [1]. However, despite the clinical success of regenerative endodontics, the type and nature of regenerated tissues are still unpredictable and uncontrollable [1].

In cell-based REPs, stem cells and growth factors are delivered via scaffolds to promote tissue regeneration [2]. This necessitates the isolation, culture, and transplantation of autologous or allogeneic stem cells onto the scaffold [3, 4]. Some of the limitations encountered with cell-based regeneration are the possibility of contamination, immunological rejection, and high expense [3]. Therefore, relying on cell homing of endogenous stem cells in clinical scenarios has gained more attention [2, 5]. Scaffolds play a crucial role in regenerative endodontics as they serve as a 3D network that promotes cellular migration, proliferation and differentiation to ultimately form new tissue [6]. Optimizing bioactive scaffolds to obtain consistent results in REPs is currently a major area of research. Decellularized ECM derived from various tissues has been studied in recent years as a scaffold for pulp regeneration [7, 8]. The creation of a decellularized scaffold that completely eliminates donor cells and antigens while keeping the ECM structure could provide an optimal and tissue specific platform for tissue regeneration [9]. These non-immunogenic and biocompatible scaffolds preserve natural intact structures, offering a particular milieu for cell population and tissue regeneration [9]. Moreover, the potential advantage of using Pulp Extracellular matrix is the tissue specificity for maintaining selected cell functions and phenotype [10]. Therefore, Decellularized bovine dental pulp-derived extracellular matrix (P-ECM) could be used as it is a natural scaffold that can best mimic the native extracellular matrix (ECM) of the pulp providing a suitable niche for organized tissue regeneration [11]. Natural components including collagen, glycosaminoglycan, growth factors, and proteoglycans are retained in the decellularized ECM scaffolds which support and facilitate cell functions offering an effective cell-homing strategy [12, 13]. Decellularized ECM scaffolds, like other collagen-based porous scaffolds, have high degradation rates, and relatively low mechanical strength, therefore they frequently require crosslinking [14].

A bovine source was selected as it is a widely available source for the preparation of the hydrogels. One major limitation of obtaining P-ECM from a xenogeneic source is potential immunogenicity [15]. However, recent evidence confirms that immunogenicity can be greatly inhibited through the use of optimal decellularization and crosslinking protocols, rendering the scaffold more biocompatible [13, 15]. Moreover, xenogeneic decellularized ECM-derived scaffolds have been known as common materials for cranio-facial regeneration in both animals and humans due to their high biocompatibility [16].

The delicate nature of the pulp tissue, the asymmetrical root canal shape, and its small volume (≈ 20 µL) all make it challenging to effectively implant pre-formed scaffolds into the root canal space [17]. Consequently, injectable scaffolds could provide a superior treatment option for dental pulp regeneration. Hydrogel scaffolds have many advantages, including maximum sterility, minimal invasiveness, the ability to fill irregular defects, and the homogeneous delivery of bioactive molecules or cells through the simple mixing of solutions before injection [17, 18]. Since hydrogels have viscoelastic characteristics that resemble soft connective tissue, they could replicate the native pulp tissue ECM by encapsulating cells and transferring nutrients and waste products and ultimately regenerate a true dentin-pulp complex [17, 19].

Hyaluronic acid (HA) hydrogels have been also suggested as potential scaffolds owing to their biocompatibility and ability to preserve extracellular spaces as well as tissue mechanical integrity [20]. Hyaluronic acid is one of the major components of the ECM. In addition, its biodegradation products have been shown to enhance angiogenesis [20, 21]. It could also serve as a controlled drug delivery vehicle by tailoring hyaluronic acid hydrogels to release growth factors or naturally occurring bioactive molecules, thereby adding a further boost to the endogenous tissue regeneration cascade [22–24]. Also, it has been approved by the FDA and the European Commission for clinical use in a lot of applications using different formulations [16]. The rationale behind the addition of hyaluronic acid to P-ECM was that it could provide more sustained protein and growth factor release characteristics of the P-ECM hydrogel by improving its viscoelastic properties as well as making it more convenient for clinical use [23].

Owing to the potential advantages of using an injectable form of dental pulp-derived ECM scaffold for dentin-pulp regeneration, the current study aimed to prepare and characterize an injectable bovine dental pulp-derived extracellular matrix (P-ECM) hydrogel. Additionally, hyaluronic acid was used as a carrier vehicle (P-ECM + HA) to optimize the physical and biochemical properties of the scaffold.

The null hypothesis of this study was that there would be no difference in the properties of hydrogels of P-ECM or P-ECM + HA combination, regarding their physical and biological characteristics.

## Methods

### Study design and ethical approval

**Fig. 1:**
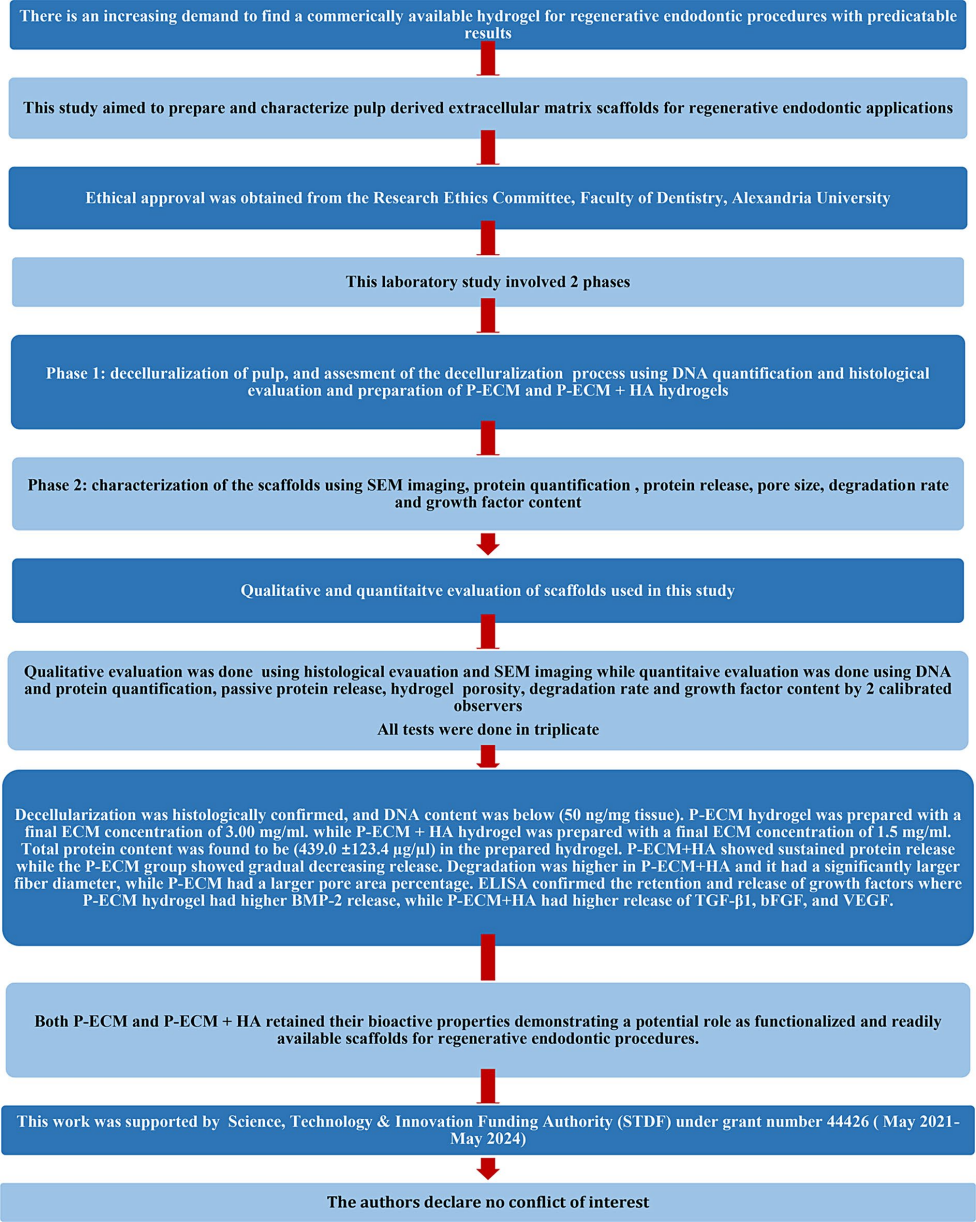
PRILE 2021 Flowchart showing the design, results and conclusions of the study

This study was done in accordance with the Preferred Reporting Items for Laboratory Studies in Endodontology (PRILE) 2021 guidelines [25] (Fig. 1). Ethical approval was obtained from the Research Ethics Committee, Faculty of Dentistry, Alexandria University, Egypt (IRB 00010556 – IORG 0008839) (0535 − 11/2022).

Preparation of samples and characterization were performed in Tissue Engineering Laboratories, Faculty of Dentistry, Alexandria University, Egypt while the scanning electron microscopic evaluation was performed in the Faculty of Science, Alexandria University, Egypt.

### Preparation of decellularized bovine pulp-derived extracellular matrix (P-ECM) hydrogels

#### Decellularization of bovine pulp tissue

**Fig. 2:**
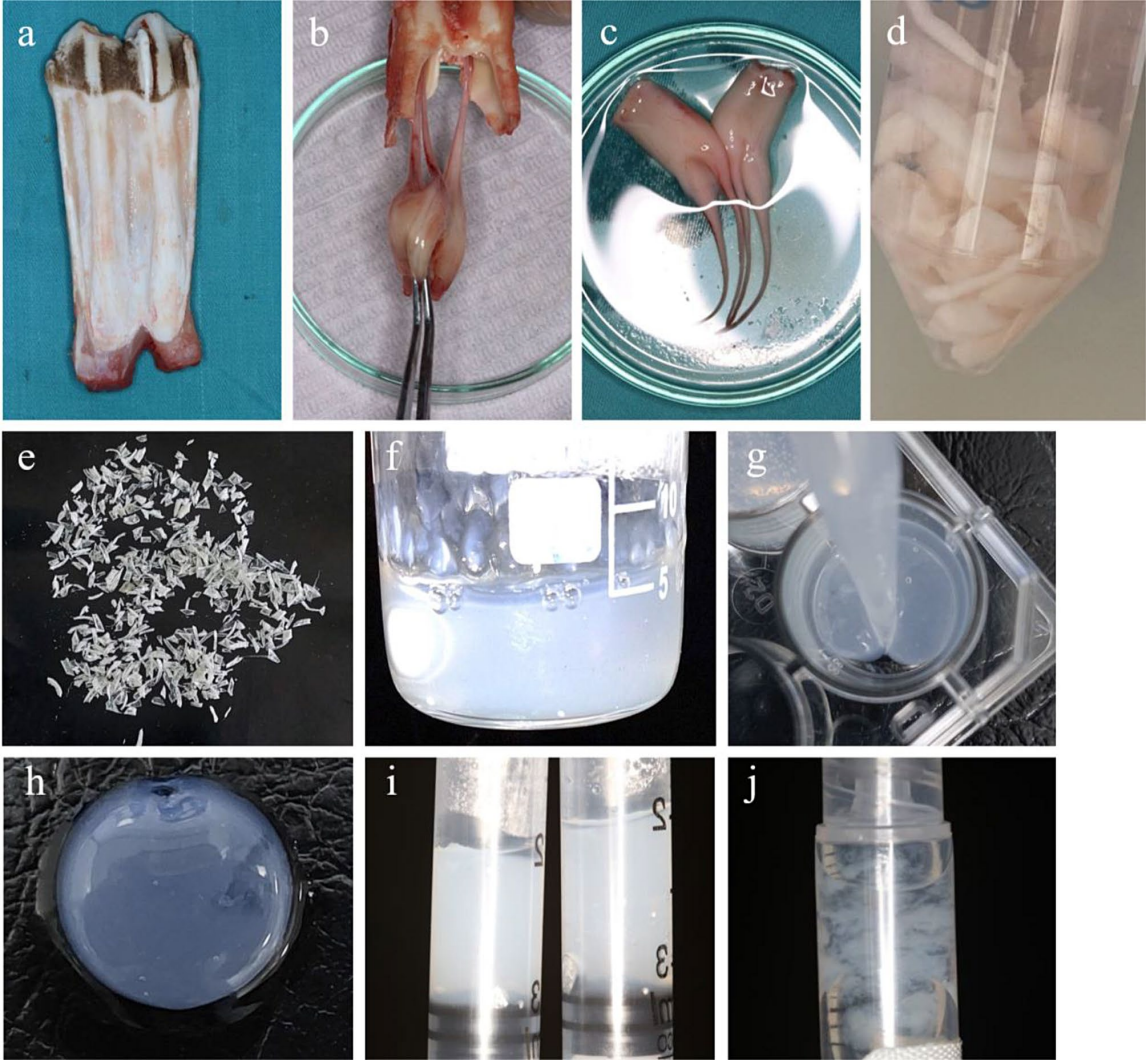
Showing (**a**) cleaned and washed bovine mandibular molar tooth; (**b**&**c**) pulp was extirpated using sterile tweezers then washed; (**d**) extirpated bovine pulp tissues were cut into small equal segments and decellularized using trypsin/ethylenediaminetetraacetic acid (EDTA), nuclease solution DNase treatment and several washes with PBS and deionized water; (**e**) lyophilized decellularized pulp was cut into small segments; (**f**, **g**&**h**) digestion of decellularized ECM to prepare P-ECM hydrogel with a final concentration of 3.00 mg/ml; (**i**)) P-ECM following sterile filtration, (**j**) physical mixing of P-ECM hydrogel with hyaluronic acid (HA) to prepare P-ECM + HA scaffold

Sample harvesting was done at a veterinary-controlled slaughterhouse (Faculty of Agriculture, Alexandria University, Alexandria, Egypt). 350–400 kg male cows (mean age 1.5-2-year-old) were slaughtered for food production purposes. Mandibular jaws were harvested, washed with sterile PBS, and immediately maintained on ice packs while being transported to the laboratory to prevent cell lysis and matrix damage. 10 Mandibular molar teeth were carefully extracted from jaws, and teeth were cracked-open. Pulp tissues were extirpated using sterile tweezers, washed with sterile phosphate buffer saline (PBS) (biowest chemicals, USA) cut into small equal segments, weighed, and kept at -40° C overnight [7] (Fig. 2).

For decellularization of pulp tissue, a 5-day decellularization protocol was performed. Samples were thawed then washed with deionized water in 4 × 15 min cycles, followed by 24 h wash with trypsin 0.05% and ethylenediaminetetraacetic acid (EDTA) 0.02% (Lonza, Basel Stücki, Switzerland). Then the samples were washed with PBS for 3 h, treated with Triton-X-100 (Loba Chemie, Mumbai, India) for 1 h then washed with PBS for 24 h. The tissue was finally treated with a nuclease solution DNase I (Enzynomics, Daejeon, Republic of Korea) for 1 h at 37° C and washed with PBS for 24 h followed by deionized water wash for another 24 h. The residual water was removed by sterile filter papers then samples were lyophilized (Heto dry winner, Thermo Scientific, USA) for 24 h and stored at -40 °C [7, 26, 27].

#### DNA quantification and histological evaluation of the decellularization

Total DNA content was quantified in native and decellularized pulp samples. DNA extraction was done using DNA extraction kit (Genetix Biotech LTD, New Delhi, India). Briefly, 25 mg of each sample was completely homogenized and dissolved in 1 ml of lysis buffer and subsequently digested with proteinase K, followed by a phenol/chloroform extraction. DNA was precipitated from the aqueous phase with 100% ethanol HPLC grade (Chemlab, Belgium), after which the extracts were subsequently washed with 70% ethanol. After DNA was dissolved in the buffer, the samples were stored at 4 °C. Two µL of DNA were used for nanodrop 2000 spectrophotometers (Thermo Fisher Scientific, Massachusetts, USA). While five µL of DNA were used for QuantiFluor dsDNA fluorescent dye (Promega, USA).

Native and decellularized pulp tissues were fixed for 24 h in 10% PBS/neutral buffered formalin solution (Elgomhoreya, Alexandria, Egypt) (pH 7.4) at 25 °C. Subsequently, samples were washed in deionized water, dehydrated in a graded ethanol series, embedded in paraffin, and dissected into 5 μm sections. The tissue slides were stained with hematoxylin and eosin (H&E), Masson’s Trichrome (MT), and Alcian blue to evaluate the cellular content, collagen content, and Glycosaminoglycans (GAGs), respectively. The stained slides were examined under a light microscope with 10x magnification (OLYMPUS CH40, Tokyo, Japan) to quantify the collagen, and GAGs contents and to confirm the absence of nuclei [7].

#### Preparation of bovine dental pulp-derived extracellular matrix (P-ECM) hydrogel and dental pulp-derived extracellular matrix combined with hyaluronic acid (P-ECM + HA) hydrogel

P-ECM Hydrogel was prepared by dissolving 10 mg pepsin (Serva, Heidelberg, Germany) in 10 mL of 0.1 M HCl. Then 30 mg of finely cut lyophilized pulp ECM were added to be digested in the prepared solution for 24 h at 4 °C, under constant stirring to obtain a final concentration of 3.00 mg/ml samples. While the mixture was on stirrer and in cold water baths, 1 M NaOH was added dropwise until pH 7 was achieved. The prepared pre-gel was then filtered using a Polyvinyl Difluoride (PVDF) hydrophilic 0.45 μm syringe filter (Labfil, Zhejiang, China) and preserved at − 40 °C [27, 28]. P-ECM hydrogel was prepared by incubating the pre-gel at 37ºC for 30 min until complete gelation is ensured. P-ECM + HA hydrogel was freshly prepared by combining 2% sterile HA solution (Restylane Lyft, Sweden) with the prepared Pulp ECM Hydrogel in equal volume (1:1) so that the final concentration of ECM in 1 ml of P-ECM was 3.00 mg while in P-ECM + HA it was 1.5 mg (Fig. 2). The P-ECM + HA hydrogel was prepared by simple physical mixing of the two hydrogels without the addition of any chemical crosslinking agents. Every ten bovine molar teeth used in this study were enough to prepare 150–170 ml of P-ECM hydrogel. Before each experiment, lyophilized pulp tissues were used to freshly prepare the required amount of hydrogels.

### Characterization of prepared P-ECM scaffolds

#### Protein quantification and release from P-ECM and P-ECM + HA hydrogels

The total protein content for hydrogels was quantified from the pre-gel solution of P-ECM using the bicinchoninic acid (BCA) assay (Biobasic, Canada) as described by Smith et al. [29]. Absorbance values were measured at 562 nm using a plate reader (TECAN, USA) and were converted to protein concentration (µg/mL) using values from the protein standard [29].

BCA protein assay was performed to determine the amount of protein that was passively released from the hydrogel following complete gelation, at predetermined time points 0, 1, 5, 14, and 28 days. As previously described, absorbance values were measured at 562 nm using plate reader then converted to protein concentration (µg/mL) using values from the protein standard [30].

#### Biodegradability of P-ECM and P-ECM + HA hydrogels

The dry weight (W0) of the scaffolds, either P-ECM or P-ECM + HA, was determined using digital sensitive balance (Sartorius, Germany). Each sample was submerged in PBS (pH 7.4) in a sealed vial. The samples were then incubated at 37 °C for 1, 5, 14, and 28 days. The scaffolds were taken out of the PBS and freeze-dried for 48 h at each time point to determine the dry weight (W1) of the scaffold that remained. The following formula was used to determine the degradation rate [31].

$${\bf{Degradation}}{\text{ }}{\bf{rate}}{\text{ }}\left( \% \right){\text{ }} = {\text{ }}\left( {{\bf{W0}} - {\bf{W1}}} \right)/{\text{ }}{\bf{W0}} \times {\text{ }}{\bf{100}}\%$$

#### P-ECM and P-ECM + HA hydrogels topology and porosity

Hydrogels were fixed in 2.5% glutaraldehyde (Electron Microscopy Sciences, Hatfield, England) for 24 h then dehydrated carefully with serial concentrations of ethanol (30, 50, 70, 80, 90, 100%). The hydrogel samples were kept in each concentration for 30 min at 4 °C. Hydrogels were then washed twice with 100% ethanol for 30 min at 4 °C, then left for 48 h until complete dryness. The hydrogels were sputter-coated with gold with a thickness of 4.5 nm. The surface topology, pore area percentage, and fiber diameter of the hydrogels were assessed using a scanning electron microscope (SEM; JSM-IT200, JEOL, Germany) at magnification 2500x, 5000x, and 10,000 × [30, 32]. Moreover, hyaluronic acid topology was identified at magnification 2500x and 5000x.

#### Growth factor release from P-ECM and P-ECM + HA

Each hydrogel was characterized for growth factor content to detect specific growth factors (TGF ß1, bFGF, BMP-2, and VEGF).

The levels of TGF-β1, bFGF, BMP-2, and VEGF growth factors were assayed in supernatants of P-ECM and P-ECM + HA hydrogels and in pre-gel of P-ECM, using enzyme-linked immunosorbent assay (ELISA) kits according to the manufacturer’s instructions [human bone morphogenetic protein 2 (BMP-2) immunoassay, R&D Systems Inc, USA]; [Transforming growth factor beta 1 (TGF-β1 ) immunoassay, Cloud Clones Corp, USA]; [ bovine basic fibroblast growth factor (bFGF) and bovine vascular endothelial growth factor (VEGF), BT-lab, China]. Absorbance values were measured at 450 nm using plate reader (TECAN, USA). The concentrations in specimens were determined from standard curves prepared from a range of known standard concentrations for each growth factor. The standards and samples were run in duplicate for both growth factors, and the average of 3 readings was used for further analysis. Results were expressed as pg/ml of pulp tissue hydrogel for TGF-β1 and BMP-2 and as ng/l for bFGF and VEGF.

#### Injectability

The injectability of pre-gel P-ECM, gelled P-ECM and P-ECM + HA were evaluated by means of universal testing machine by using commercially available luer-lock syringes of 3 mL with a needle of diameter of 22 gauge. The hydrogels were added then the plungers of needles were added and positioned between the compression plates of a Universal Testing Machine. Then, the prepared hydrogel was extruded by applying a compression rate of 1 mm per minute up to a maximum force of 5 N for 2 min [33–35]. The injectability coefficient was calculated as the weight% of the hydrogel that was extruded from the syringe relative to the total mass of hydrogel initially placed in the syringe (supplementary Fig. 1).

### Statistical analysis

The study was carried out using triplicate samples. Each experiment was carried out 3 times (*n* = 3) in triplicates for each group to assess the characterization of P-ECM and P-ECM + HA in terms of DNA and protein quantification, passive protein release, hydrogel topology and porosity, degradation rate, injectability and growth factor content. Data were collected by two calibrated observers and presented as mean and standard deviation. The normality of all measured outcomes was tested using the Shapiro Wilk test and Q-Q plots. Normal distribution was confirmed for DNA quantification, protein release, fiber diameter, and porosity while other outcomes were not normally distributed. Comparison between groups was done using an independent t-test or Mann Whitney U test. Repeated measures ANOVA was performed to assess the effect of time and intervention on protein release, while the Friedman test followed by post hoc test with Bonferroni correction was used to assess changes across time regarding all other outcomes within each group. All tests were two tailed and the significance level was set at p-value ≤ 0.05. Data were analyzed using IBM SPSS for Windows version 23, Armonk, NY, USA. Graphical presentation was done using GraphPad Prism version 10.0.0 for Windows, GraphPad Software, Boston, Massachusetts USA. Page 3.

## Results

### DNA quantification and histological evaluation of decellularization

**Fig. 3:**
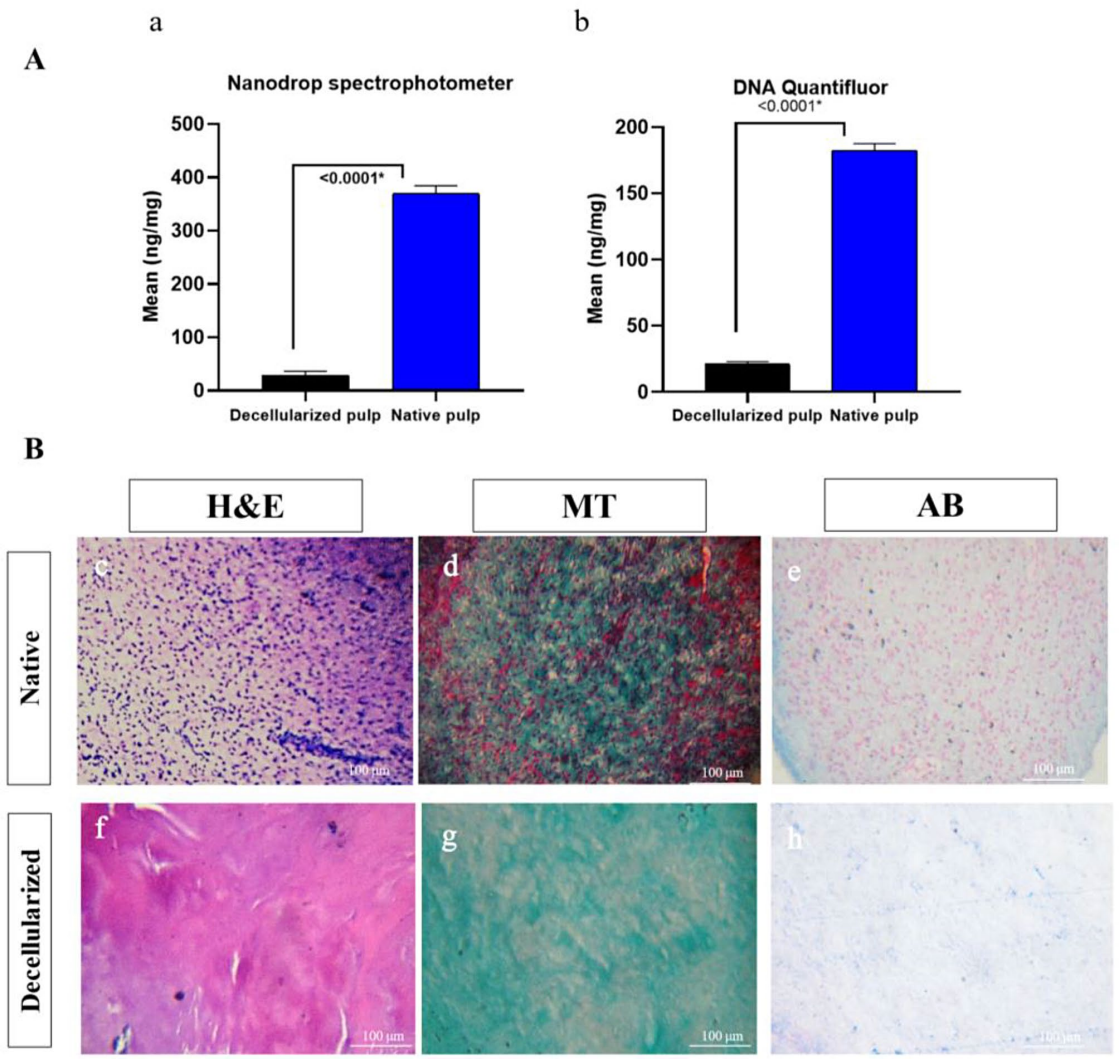
Quantitative and qualitative assessment of decellularization; (**A**) quantitative assessment of decellularization; (**a**) Nanodrop spectrophotometer quantification showing the difference in values between decellularized pulp and native pulp showing a significant difference (*p* < 0.0001); (**b**) QuantiFluor dsDNA fluorescent dye showing a significant difference (*p* < 0.0001) between DNA content of native and decellularized pulp; (**B**) Qualitative assessment using histological evaluation of decellularized pulp; (**c**) native pulp at 10x magnification stained with H&E; (**d**) Native pulp at 10x magnification stained with Masson’s Trichrome (MT) stain; (**e**) native pulp at 10x magnification stained with Alcian Blue (AB) stain; (**f**, **g**&**h**) showing decellularized P-ECM at 10x magnification stained with H&E, MT and AB, respectively. The absence of nuclei is evident in all decellularized sections. Collagen bundles (stained blue by MT stain) and tissue architecture were preserved following decellularization. GAGs content (stained faint blue by AB) is evident in both

Bovine pulp tissues were successfully decellularized. DNA was found to be below the cut-off point (50 ng/mg tissue) using Nanodrop spectrophotometer (28.23 ± 7.87 ng/mg) in comparison to native tissues (369.5 ± 14.78 ng/mg) (*P* < 0.0001) and using Quantifluor dsDNA dye fluorescent (21.18 ± 1.75 ng/mg tissue) in comparison to native tissues (182.4 ± 5.19ng/mg) (*P* < 0.0001) (Fig. 3).

Histological examination of native and decellularized bovine pulp samples stained with hematoxylin and eosin (H&E), Masson’s Trichrome (MT), and Alcian blue staining revealed the absence of nuclei, retention of collagen content, and glycosaminoglycans (GAGs), respectively (Fig. 3).

### Characterization of prepared P-ECM scaffolds

#### Protein quantification and release from P-ECM hydrogels

**Fig. 4:**
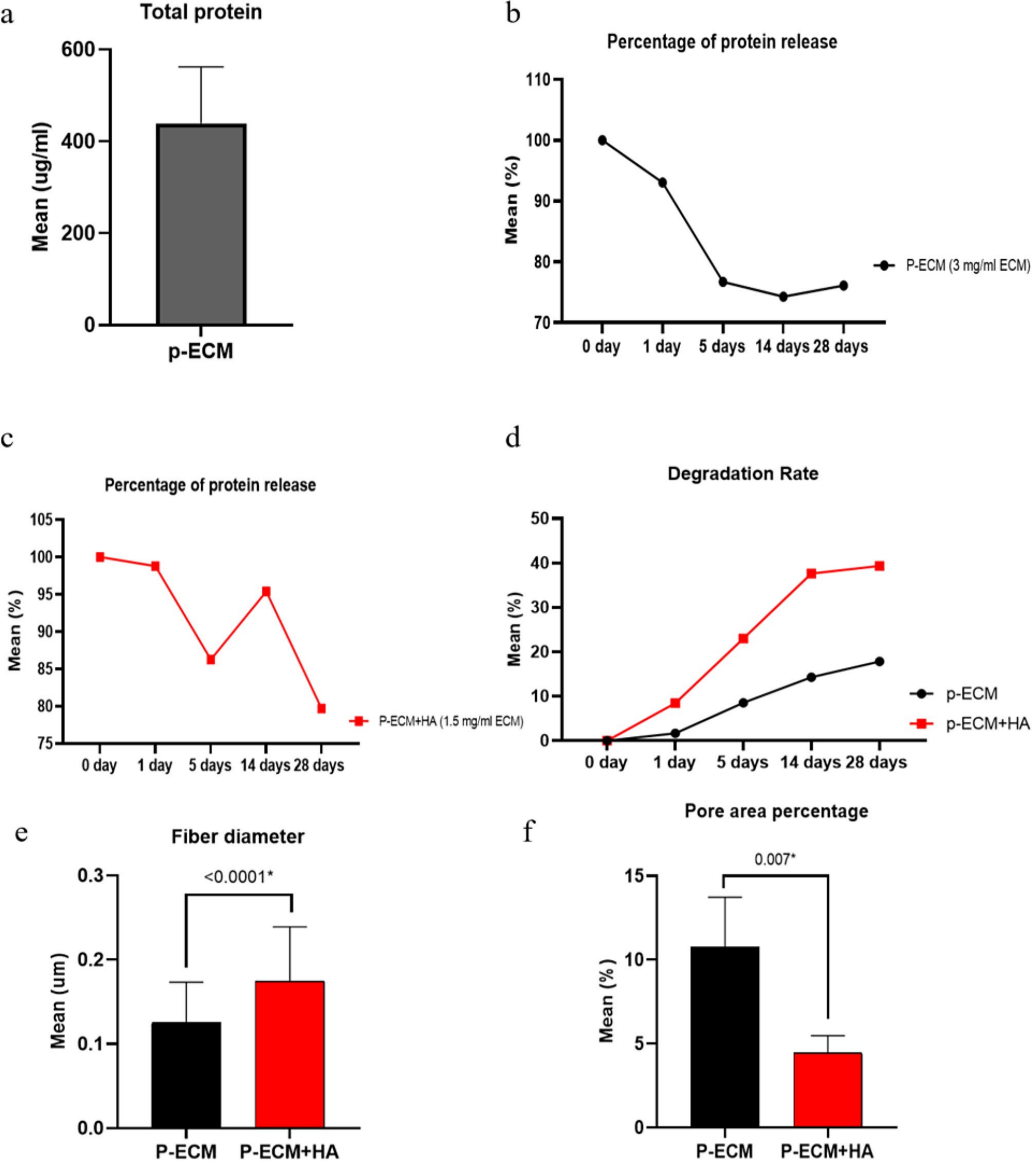
Characterization of hydrogels; (**a**) total protein content of P-ECM pre-gel; (**b**) percentage of protein release of P-ECM showing burst release at 0 D then decreasing release till reaching 28 days where release percentage at 28 days is slightly higher than 14 days (**c**) percentage of protein release of P-ECM + HA showing burst release at 0 D then decreasing release till reaching day 5 then increase at day 14 then decrease again at day 28; (**d**) biodegradation rate of hydrogels showing increase percentage for P-ECM + HA group; bar graph showing a comparison between P-ECM and P-ECM + HA regarding (**e**) fiber diameter and (**f**) pore area percentage where fiber diameter of P-ECM + HA was larger than P-ECM (*p* < 0.0001) which reflects on the larger pore area percentage of P-ECM was larger than P-ECM + HA (*p* < 0.007*)

The total protein content for P-ECM hydrogel was found to be (439.0 ± 123.4 µg/ml) (Fig. 4).

Passive protein release was quantified for both groups P-ECM and P-ECM + HA for 5-time points over 28 days. P-ECM showed significantly higher values at 0 days (147.56 ± 6.29 µg/mL, 88.03 ± 7.99 µg/mL, respectively) (*p* < 0.0001). P-ECM showed higher mean scores at 1 day-time point (138.21 ± 35.03 µg/mL, 88.26 ± 28.97 µg/mL, respectively), at 5 days’ time point (114.04 ± 30.75 µg/mL, 76.71 ± 20.02 µg/mL, respectively), and at 14 days (109.79 ± 13.12 µg/mL, 86.02 ± 45.33 µg/mL, respectively). After 28-days P-ECM showed significantly higher values (112.58 ± 13.32 µg/mL, 69.90 ± 6.52 µg/mL, respectively) (*p* < 0.0001) (Table 1). Both time and the scaffold composition significantly affected the rate of protein release (supplementary file 1, Table 1). However, the amount of protein released was significant only between day 0 and day 28 as well as between day 1 and day 5 for both groups (supplementary file 1, Table 2). The concentration of ECM in the hydrogel was 3 mg/mL in P-ECM hydrogel while in P-ECM + HA hydrogel it was 1.5 mg/mL.

The percentage of protein release was measured as follows; the 0 day time point was considered as baseline and considered 100%. The rest of the time points were compared to the 0 day to represent the percentage from the 0 day. So the 100% of the zero day represented the baseline. There was a descending release throughout the 28 days. However the lowest release percentage in the 28 days was approximately 75% in the P-ECM and approximately 80% in the P-ECM + HA thus there was still protein release till the 28 days time point.(Fig. 4).

**Table 1:** Comparison of protein release (ug/ml) between P-ECM and P-ECM + HA hydrogels

	*P*-ECM	*P*-ECM + HA	*p*-value
0 day	147.56 ± 6.29	88.03 ± 7.99	< 0.0001*
1 day	138.21 ± 35.03	88.26 ± 28.97	0.070
5 days	114.04 ± 30.75	76.71 ± 20.02	0.088
14 days	109.79 ± 13.12	86.02 ± 45.33	0.353
28 days	112.58 ± 13.32	69.90 ± 6.52	0.001*

Data is presented as Mean ± SD

*Statistically significant difference at *p* value ≤ 0.05

#### Biodegradability of P-ECM and P-ECM + HA hydrogels

The biodegradability rate was measured, and it showed slight degradation over time in the P-ECM group while P-ECM + HA group showed marked declination over 28 days duration (Fig. 4). P-ECM had a starting percentage of (0.007 ± 0.010%) at day 0 which increased slightly to (1.697 ± 2.381%) on day 1 then increased again at day 5 to reach (8.574 ± 12.109%) then (14.324 ± 20.243%) on day 14 to reach its peak on day 28 (17.845 ± 25.224%) while for P-ECM + HA it had a starting percentage of (0.035 ± 0.049%) at day 0 which increased slightly to (8.470 ± 11.897%) on day 1 then increase again at day 5 to reach (23.035 ± 32.523%) then (37.635 ± 53.199%) on day 14 to reach its peak on day 28 (39.365 ± 55.650%).

#### Hydrogel topology and porosity

**Fig. 5:**
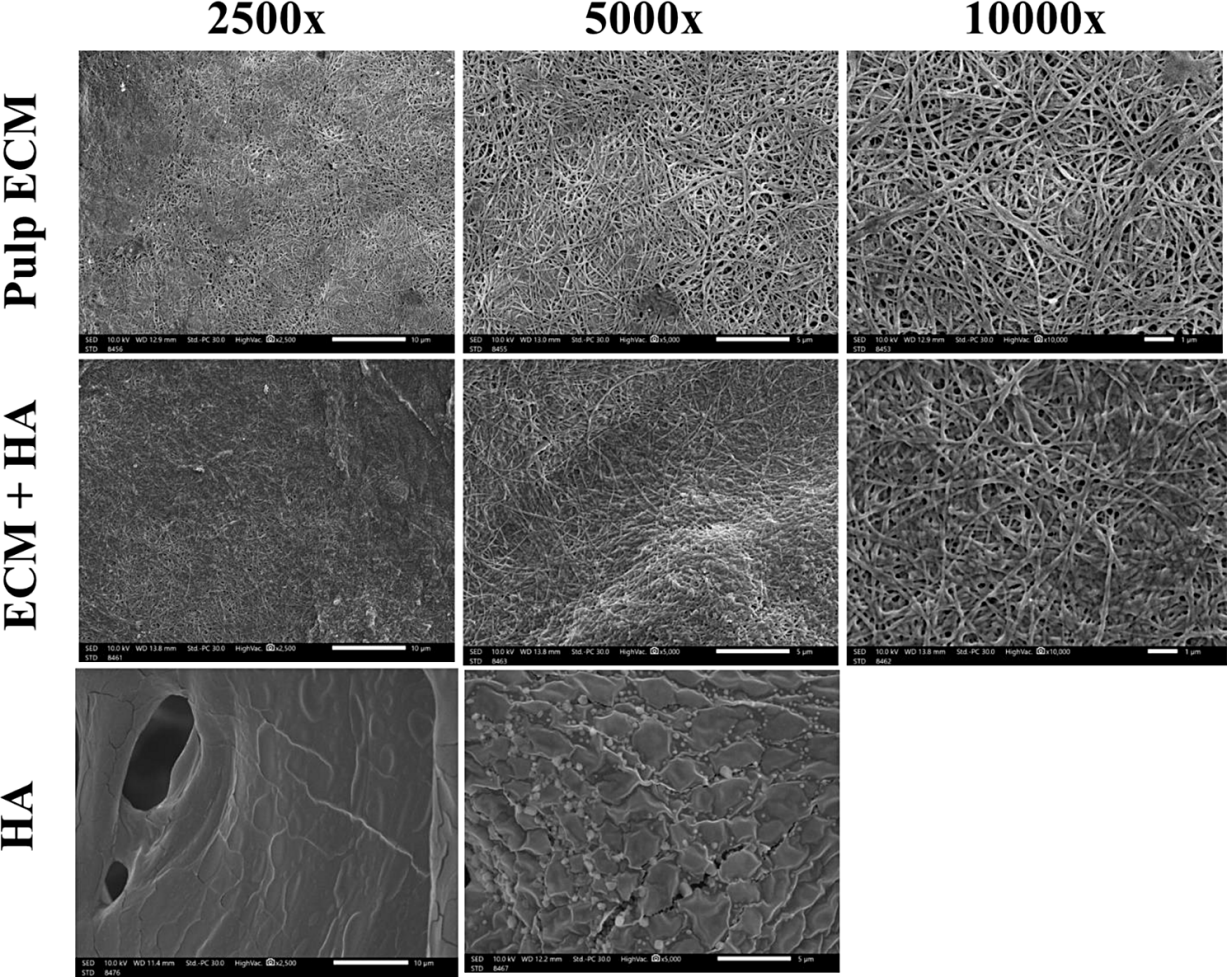
SEM analysis of hydrogels; showing a comparison between P-ECM, P-ECM + HA hydrogels & HA showing the presence of fibers and pores and intermingling structure in the P-ECM, P-ECM + HA hydrogels and the pore-less structure of HA

SEM analysis for P-ECM and P-ECM + HA hydrogels showed their architecture, presence of intermingling fibers, and pores in both groups. Moreover, hyaluronic acid topology was identified showing pore-less structure (Fig. 5). Fiber diameter for both groups was identified in the SEM images using ImageJ software [36]. The diameter in P-ECM was (0.13 ± 0.05 μm) while in P-ECM + HA group was (0.17 ± 0.06 μm) showing significantly larger fiber diameter in the latter (*p* < 0.0001) (Fig. 4). While pore area percentage in P-ECM group (10.76 ± 2.97%) was significantly larger compared to P-ECM + HA ( 4.44 ± 1.04% ) (*p* < 0.007*) (Fig. 4).

#### Growth factor release from P-ECM and P-ECM + HA hydrogels

Each hydrogel was characterized for the growth factor content using an enzyme linked immunosorbent assay (ELISA) to detect angiogenic and osteogenic growth factors. Namely, TGF-β1, bFGF, BMP-2, and VEGF. The concentration of ECM in the hydrogel was 3 mg/ml for P-ECM while it was 1.5 mg/ml for P-ECM + HA.

##### **TGF-β1**

TGF-β1 was detectable in both groups for 5-time points over 28 days (0, 1, 5, 14, 28 days). P-ECM + HA showed higher mean scores on day 0 (165.46 ± 20.81 pg/ml). On the contrary, on days 1 and 5 P-ECM showed higher mean scores (141.79 ± 30.90 pg/ml and 112.49 ± 21.43 pg/ml, respectively). On days 14 and 28, P-ECM + HA showed higher mean scores (128.26 ± 24.85 pg/ml and 133.53 ± 3.79 pg/ml, respectively). It is worth mentioning that TGF-β1 expression in P-ECM peaks at day 0 and gradually decreases till day 28. On the other hand, expression of TGF-β1 in P-ECM + HA peaks at day 0 and gradually decreases till day 5 then gradually increases till day 28. As illustrated in (Table 2).

**Fig. 6:**
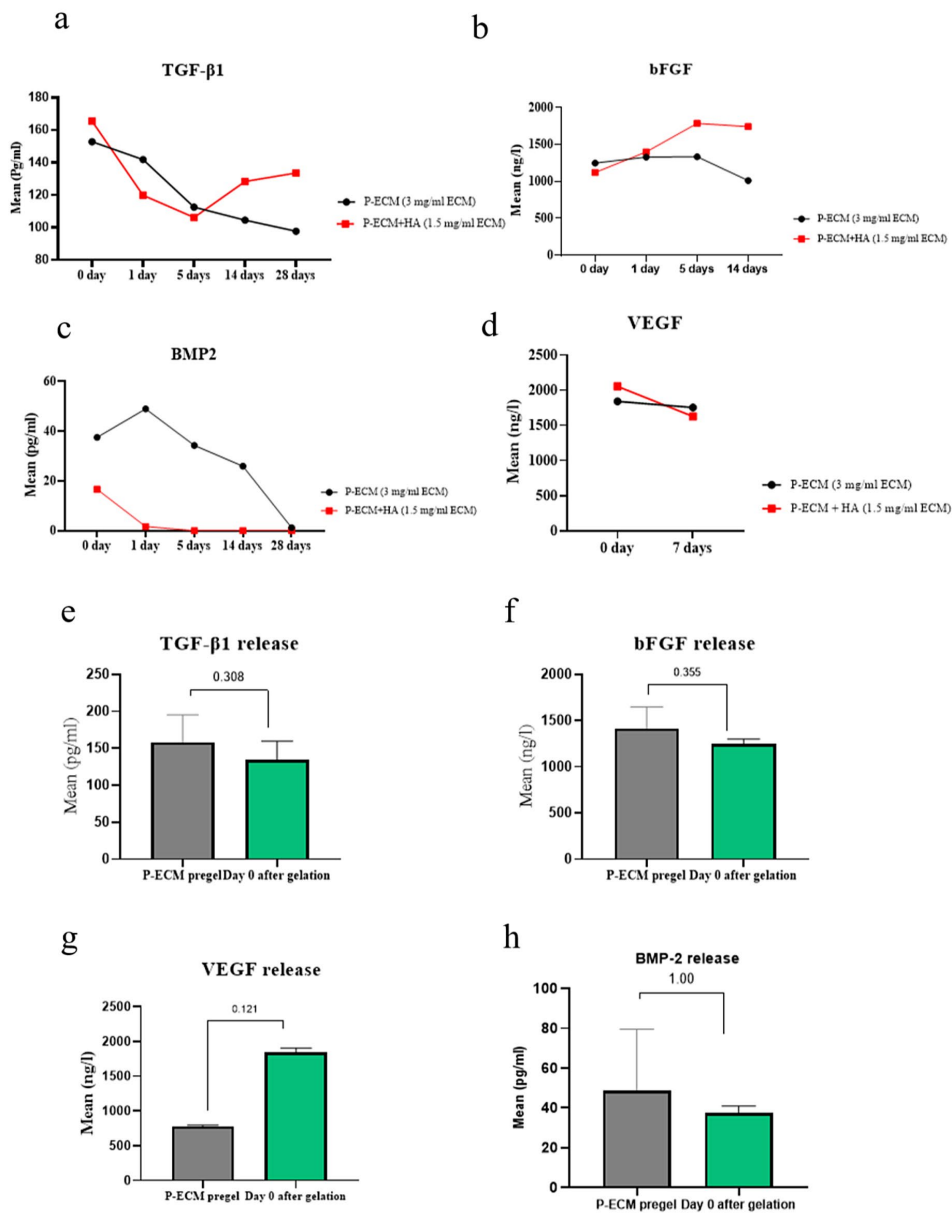
Growth factor content; (**a**) Comparison between P-ECM & P-ECM + HA hydrogels regarding TGF-β1 release showing increased release in P-ECM + HA at 0,14 and 28 days ; (**b**) bFGF release showing higher release in P-ECM + HA group in 1,5 and 14 days ; (**c**) BMP2 release showing nearly indetectable concentration in P -ECM + HA while detectable in P-ECM; (**d**) VEGF release showing increased release of P-ECM at 7 days timepoint (**e**, **f**, **g**&**h**) bar graph showing comparison between P-ECM pre-gel and zero time point hydrogel after gelation

There was a substantially higher release by P-ECM + HA relative to P-ECM in 0, 14 and 28 days by considering the release of TGF-β1 in units of pg/ml (Fig. 6).

Also, a comparison was made between P-ECM pre-gel and zero time point of P-ECM after gelation showing higher mean scores for P-ECM pre-gel (Fig. 6).

**Table 2:** Comparison of TGF-β1 (pg/ml) between P-ECM and P-ECM + HA hydrogel

	*P*-ECM	*P*-ECM + HA	*P* value
0 day	158.33 ± 36.76	165.46 ± 20.81	0.564
1 day	141.79 ± 30.90	119.77 ± 4.08	0.355
5 days	112.49 ± 21.43	106.15 ± 1.39	1.00
14 days	104.46 ± 35.58	128.26 ± 24.85	0.248
28 days	97.62 ± 0.36	133.53 ± 3.79	0.121
***P*** **value**	0.267	0.147	

Data is presented as Mean ± SD

*Statistically significant different at *p* value ≤ 0.05

##### **bFGF**

bFGF was detectable in both groups for 4 time points over 14 days (0, 1, 5, 14 days). P-ECM showed higher mean scores on day 0 (1249.05 ± 50.68 ng/l). On the contrary, on days 1,5 and 14 P-ECM + HA showed higher mean scores (1400.99 ± 256.58 ng/l, 1787.70 ± 63.56 ng/l and 1744.08 ± 67.04 ng/l, respectively). It is worth mentioning that bFGF expression in P-ECM and P-ECM + HA gradually increases till day 5 and then decreases on day 14. However, the release of bFGF in terms of ng/l was more in P-ECM + HA (Fig. 6) (Table 3).

Also, a comparison was made between P-ECM pre-gel and zero time point of p-ECM after gelation showing higher mean scores for P-ECM pre-gel (Fig. 6).

**Table 3:** Comparison of bFGF (ng/l) between P-ECM and P-ECM + HA hydrogel

	*P*-ECM	*P*-ECM + HA	*p*-value
0 day	1249.05 ± 50.68	1122.91 ± 131.18	0.439
1 day	1330.14 ± 117.57	1400.99 ± 256.58	1.00
5 days	1336.49 ± 147.98	1787.70 ± 63.56	0.121
14 days	1012.24 ± 5.65	1744.08 ± 67.04	0.121
***p-value***	0.241	0.145	

Data is presented as Mean ± SD

*Statistically significant different at p value ≤ 0.05

##### **BMP-2**

BMP-2 was measured in both groups for 5-time points along 28 days (0, 1, 5, 14, 28 days). It was only detectable in the P-ECM + HA group at 0&1 days, while on the other time points, it had negligible expression. While in the P-ECM group, it increases gradually from 0 to 1 day peaking at 1 day, then it decreases gradually till the 28 days time point (Table 4) (Fig. 6).

Also, a comparison was made between P-ECM pre-gel and zero time point of p-ECM after gelation showing higher mean scores for P-ECM pre-gel (Fig. 6).

**Table 4:** Comparison of BMP-2 (pg/ml) between P-ECM and P-ECM + HA hydrogel

	*P*-ECM	*P*-ECM + HA	*p* value
0 day	37.56 ± 3.44	16.65 ± 1.51	0.121
1 day	49.03 ± 8.76	1.68 ± 2.38	0.121
5 days	34.30 ± 6.39	0.0 ± 0.0 (undetectable)	0.102
14 days	25.99 ± 2.82	0.0 ± 0.0 (undetectable)	0.102
28 days	1.25 ± 1.76	0.0 ± 0.0 (undetectable)	0.317
***p*** **value**	0.107	0.132	

Data is presented as Mean ± SD

*Statistically significant different at *p* value ≤ 0.05

##### **VEGF**

VEGF was detectable in both groups for 2 time points over 7 days (0 and 7 days). The highest release was detected at the 0-day time point in both groups, where P-ECM + HA showed the highest initial release (2052.40 ± 108.05 ng/l). While at the 7-day time point, P-ECM showed higher release (1753.00 ± 59.40 ng/l) (Table 5) (Fig. 6).

Also, a comparison was made between p-ECM pre-gel and zero time point of p-ECM after gelation showing higher mean scores for zero time point of P-ECM after gelation (Fig. 6).

**Table 5:** Comparison of VEGF (ng/l) between P-ECM and P-ECM + HA hydrogel

	*P*-ECM	*P*-ECM + HA	*p*-value
0 day	1839.90 ± 68.31	2052.40 ± 108.05	0.121
7 days	1753.00 ± 59.40	1625.40 ± 449.72	1.00
***p-value***	0.655	0.180	

Data is presented as Mean ± SD

*Statistically significant different at *p* value ≤ 0.05

### Injectability

All hydrogels were injectable. Injectability coefficient %was determined. P-ECM pre-gel had the highest injectability coefficient (69.27 ± 0.94%), followed by the gelled P-ECM (62.59 ± 0.43%) then the least injectability coefficient, was for the P-ECM + HA group (59.75 ± 0.45%). Significant differences were found between the groups (supplementary Fig. 2).

## Discussion

Regenerative endodontic procedures are becoming more feasible in everyday clinical scenarios. However, optimizing a bioactive scaffold that could recapitulate true dentin-pulp regeneration as a cell-homing approach is still a critical area of research [2]. One of the crucial factors of tissue regeneration is the selection of an appropriate scaffold. Ideal scaffolds should be biocompatible, preserve native structure, and promote tissue regeneration rather than repair. Hydrogels provide a clinically applicable ready-made material that conforms to irregular canal shapes. Compared to the pre-formed scaffold, the injectable scaffold has several advantages, including injectability in a minimally invasive manner with a homogenous concentration and with greater ease. Considering the size, morphology, and complicated structure of dental and craniofacial tissues, an injectable scaffold is more appealing than a pre-formed one. For example, the root canal is a long, narrow channel with an average total volume of approximately 20 µl [20, 37]. The use of ECM in the form of hydrogel has recently gained much attention and has been studied for the previous reasons. It was demonstrated that the physical properties and stiffness of scaffolds used in pulp regeneration could influence the nature of regenerated tissues [38]. Therefore, the use of a hydrogel form with a relatively low stiffness might promote the formation of vascularized pulp-like tissues rather than the induction of hard tissue formation [39, 40]. The influence of using the prepared hydrogel scaffolds on stem cell characteristics as well as potential in vivo applications still needs to be evaluated in future studies.

In this study, a naturally derived biomimetic P-ECM scaffold was prepared from a widely available bovine source. This could offer tissue specific endodontic regeneration as it provides a natural microenvironment for recruited stem cells [41–43]. However, being prepared from a bovine source, optimal decellularization must be ensured to avoid possible immunogenicity without altering the structure and bioactive properties of the scaffold [8, 9, 41, 44]. In this study, a 5 days decellularization protocol was chosen following the protocol described by Bakhtiar et al. [7]. However, modifications were made in the trypsin/EDTA concentration, the use of deionized water and PBS washes based on a decellularization pilot study. In this decellularization protocol, sodium dodecyl sulfate (SDS) was not used as several studies showed that it results in collagen degradation and residual toxicity in the tissues [27, 45]. The residual DNA content of the extracellular matrix is thought to be the main source of antigenicity that could provoke a host immunological response [46]. However, it has been reported that there is no significant host response to decellularized ECM containing 50 ng/mg of DNA or less [15]. Therefore, DNase treatment followed by several washes with deionized water and PBS were made to ensure that residual DNA was below the cut-off point. Indeed, DNA content was found to be significantly less following decellularization in comparison to native tissues, which was consistent with Alqahtani et al. [11], Bakhtiar et al. [27] and Lee et al. [47]. In addition, qualitative histological assessment confirmed the absence of nuclei in decellularized pulp, retention of glycosaminoglycans (GAGs), and collagen content which was in accordance with the findings reported by Elwerfelli et al. [8] and Song et al. [48]. In fact, it was reported that tissue collagen and GAGs play important roles in the regulation of cell-ECM interaction and subsequent intercellular processes. They also contain matrix-bound adhesion/growth factors, which are crucial for the regeneration process [49].

In this study, a concentration of 3.00 mg/ml of bovine dental pulp-derived ECM, prepared in a hydrogel form, was used according to a study by Bakhtiar et al. who reported that this concentration had optimal physical and biological properties [7]. Being an injectable hydrogel may provide a clinically applicable ready-made material that conforms to irregular canal shapes [19, 50]. For the preparation of a novel ECM + HA hydrogel, physical mixing of gelled P-ECM with HA hydrogel was done in a 1:1 ratio. This novel combination was thought to alter the properties of the hydrogel by having a sustained release of growth factors [20, 24, 50, 51].

One of the major advantages of developing an ECM-based hydrogel is that natural physical crosslinking takes place which transforms the ECM pre-gel solution into a well-formed hydrogel. This process is associated with ECM solubilization by enzymatic (pepsin) digestion. Pepsin digests ECM into several incompletely digested proteins, proteoglycans, growth factors, and matricellular proteins, including collagens, elastin, laminin, fibronectin, hyaluronan, heparan, basic fibroblast growth factor (bFGF), vascular endothelial growth factor (VEGF), insulin-like growth factor (IGF), transforming growth factor (TGF)-β, tenascin, osteopontin, and thrombospondin. Among them, a major component of ECM is collagen, which is involved in processes of cell growth, proliferation, migration and differentiation through inducing cell signalization and has attractive characteristics. It is polymerized by the self-assembly process of the fibrillar structure to generate a gel at body temperature (37 °C) and neutral pH (7.4). The collagen fiber cross-linking is promoted by hydrophobic and electrostatic interactions. This temperature- and pH-dependent sol–gel state of collagen in ECM allows an injectable ECM gel [52]. The P-ECM + HA hydrogel in the current study was formed by physical mixing of gelled P-ECM and hyaluronic acid with a 1:1 concentration with no addition of chemical cross-linkers [51].

Results of injectability testing showed that injectability coefficient changed from the pre-gel to gel state confirming gelation. Moreover, the addition of HA significantly decreased the injectability coefficient of gels. However, further investigation of different concentrations of P-ECM could be tested to assess alterations in injectability. Furthermore, chemical cross-linking has been suggested to circumvent some of these limitations [53]. In this study the total protein content in the P-ECM hydrogel was measured. Moreover, both hydrogels showed continuous protein release over a period of 28 days [54–56].Both Hydrogels had a burst release at 0 day time point. P-ECM hydrogel showed higher protein release which continued to gradually decrease over time. While P-ECM + HA showed a greater retention of proteins throughout the timepoints. This might be due to physical mixing with HA which might alter the degree of release of growth factors and proteins having a more sustained release [51]. It is important to note that in this study, the 0 day time point was considered as 100% release to which the release of all consecutive time points was compared. While comparisons could have been made with the pregel which would hypothetically contain the total amount of proteins and growth factors, this was not done for two reasons. The first is that the suggested use of these hydrogels is to be injected as pre-formed hydrogels in the root canal and not as pregel solutions for ease of application and to allow retention of the hydrogels in the irregular root canal spaces. The second reason is that not all the proteins present in the hydrogels are guaranteed to be released as some proteins are matrix-bound. Hence, their detection in the pregel does not necessarily mean that they are expressed when the hydrogel is formed [37, 57, 58].

In REPs, the ideal rate of scaffold degradation is thought to be from 6 to 8 weeks in order to allow for remodeling and replacement by de-novo tissues and to sustain the turnover of inflammation and regeneration [59, 60]. In this study, it was found that P-ECM + HA had more degradation rate percentage than P-ECM. This could be explained by the fact that the ECM in the combination group was crushed during mixing exposing more surface area to be biodegraded. It should also be noted that the addition of HA may have led to increased initial weight of the scaffold. This was in accordance with that described by Bakhtiar et al. who reported a similar rate of degradation [7, 59].

Moreover, hydrogel topology revealed the presence of pores, and intermingling of fibers after decellularization which is due to the ECM component which was in accordance with that described by Elwerfelli et al. and Yuanyuan Shi et al. [8, 9, 61]. However, P-ECM + HA had a larger fiber diameter, smaller pore area percentage, and more dense structure, which might be due to mixing with HA [59].

In REPs, the presence and severity of inflammation may hinder the release of biological markers that are sequestered in the dentine matrix which are released by conditioning agents before eliciting apical bleeding [62]. Therefore, relying on naturally occurring tissue-specific growth factors in the prepared scaffolds could be valuable. Therefore, growth factors that are present in the native pulp and essential for regenerative process, were quantified. TGF-β1 and bFGF are known to stimulate cell migration and contribute to cell proliferation and differentiation. Moreover, VEGF is crucial for neovascularization and enhancement of angiogenesis. While BMP-2 promotes odontoblastic differentiation and dentin formation [11, 63, 64].

In this research, the release pattern of the forementioned growth factors was detected in the supernatants of both groups after gelation. TGF-β1, bFGF and VEGF were released from both hydrogels which was in accordance with the results of Alqahtani et al., where VEGF and TGF-β1 were detected after decellularization of pulp tissues. However, in their study, bFGF was not detected which may be due to different decellularization and sterilization protocols [11]. VEGF showed almost sustained release up to 7 days which could be the crucial time during natural wound healing cascade [65, 66].

Addition of hyaluronic acid to P-ECM seems to enhance and modify its’ release characteristics in terms of growth factors and proteins. This was in accordance with the findings reported by Lio et al. [24]. Although P-ECM + HA had only half the concentration of the ECM, both P-ECM and P-ECM + HA hydrogels showed comparable results, the P-ECM + HA had a larger fiber diameter, more dense structure and sustained growth factor release of TGF-β1, bFGF, and VEGF. This could be attributed to the physical nature of the combined P-ECM + HA scaffold having an increased density and decreased pore size which could explain the longer retention and sustained release of growth factors [24]. The growth factor release at day 0 after gelation is comparable to that of native intact and inflamed pulp of other studies in which TGF-β1 had almost half amount that secreted by intact pulp and higher VEGF and bFGF release than intact pulp [67, 68]. P-ECM hydrogel BMP-2 release at day 0 was in accordance with Jugoslav et al. in which it had less release than intact pulp but more release than injured pulp after indirect pulp capping (IPC) [69]. While, P-ECM + HA BMP-2 release at day 0 had similar amount of release of injured pulp after IPC [69].

BMP-2 was detectable with low levels in P-ECM + HA in 0 and 1 days while it was undetectable in the rest of time points which may be due to affinity of hyaluronic acid to BMP-2 [70]. Therefore it may need more tailoring to have a sustained BMP-2 release. In contrast, it was detectable in P-ECM in the 5 time points showing increasing release from 0 to 1 days then descending release till reaching 28 days.

Further research is still needed to assess other important growth factors related to dentin-pulp regeneration. Different concentrations of P-ECM and HA with different molecular weights should be compared. Moreover, the direct effects of the prepared hydrogels on stem cell functions should be evaluated paving the way for future in vivo applications. Despite the analysis of protein and different growth factors release, one of the limitations of the current study is that the functional activity of these scaffold hydrogels was not assessed. This would require performing extensive cellular assays as well as in vivo animal studies. Moreover, cumulative protein and growth factor release is recommended for future applications. Another additional limitation is that, due to the biological nature of these scaffolds, inherent variability in the results may be expected. However, this was overcome in the current study by pooling a large number of bovine pulps prior to initiating the processing.

Within the limitations of this study, it was concluded that a hydrogel form of decellularized P-ECM retained its architecture, collagen and protein content. P-ECM hydrogel had a larger pore area percentage, better rate of degradation, protein release, higher injectability coefficient and BMP-2 release. The novel combination of P-ECM + HA had a larger fiber diameter, more dense structure and sustained growth factor release of TGF-β1, bFGF, and VEGF. These results highlight the promising potential of the prepared P-ECM-based scaffolds for further studies and comparison with other biomaterials in regenerative endodontic applications.

## Conclusions

Bovine decellularized pulp ECM could serve as a readily available affordable injectable hydrogel pulp regeneration. Both P-ECM and P-ECM + HA retained their bioactive properties demonstrating a potential role as functionalized and commercial scaffolds for regenerative endodontic procedures.

## Electronic supplementary material

Below is the link to the electronic supplementary material.

Supplementary Material 1

Supplementary Material 2

## Data Availability

The datasets used and/or analysed during the current study are available from the corresponding author on reasonable request.

## Abbreviations

ANOVA: Analysis of variance
BCA: bicinchoninic acid assay
BCA: Bicinchoninic acid assay
bFGF: Basic fibroblast growth factor
BMP-2: Bone morphogenrtic protein
ECM: extracellular matrix
EDTA: ethylenediaminetetraacetic acid
H&E: hematoxylin and eosin
HA: Hyaluronic Acid
IPC: Indirect pulp capping
PBS: Phosphate buffered saline
P-ECM: pulp-derived extracellular matrix
P-ECM + HA: pulp-derived extracellular matrix/Hyaluronic Acid Combination
REPs: regenerative endodontic procedures
SDS: Sodium dodecyl sulfate
SEM: Scanning electron microscope
TGF-β1: Transforming growth factor beta 1
VEGF: Vascular endothelial growth factor
