# Supplementary material for: Preparation and characterization of bovine dental pulp-derived extracellular matrix hydrogel for regenerative endodontic applications: an in vitro study
Source: BMC Oral Health. 2024 Oct 24;24:1281. doi: 10.1186/s12903-024-05004-z (PMC11515367; doi:10.1186/s12903-024-05004-z)
Supplement: Supplementary file 2 — Supplementary Material 2 [file 12903_2024_5004_MOESM2_ESM.docx]

**Supplementary table 1: Repeated Measures ANOVA assessing the effect of material and time on** **protein release in both groups.**

| **Variables** | **Mean Square** | **F test** | ***p-value*** | **Ƞ^2^** |
| --- | --- | --- | --- | --- |
| Time | 2145.10 | 4.45 | 0.035* | 0.426 |
| Materials | 18189.49 | 9.10 | 0.024* | 0.603 |
| Time x materials | 710.35 | 1.47 | 0.247 | 0.197 |

**Supplementary table 2: Pairwise comparisons regarding protein release in both groups.**

|  | Groups | Compared to | *p-value* |
| --- | --- | --- | --- |
| Time | 0 day | 1 day | 1.00 |
|  |  | 5 days | 0.176 |
|  |  | 14 days | 0.922 |
|  |  | 28 days | 0.001* |
|  | 1 day | 5 days | 0.012* |
|  |  | 14 days | 1.00 |
|  |  | 28 days | 0.411 |
|  | 5 days | 14 days | 1.00 |
|  |  | 28 days | 1.00 |
|  | 14 days | 28 days | 1.00 |


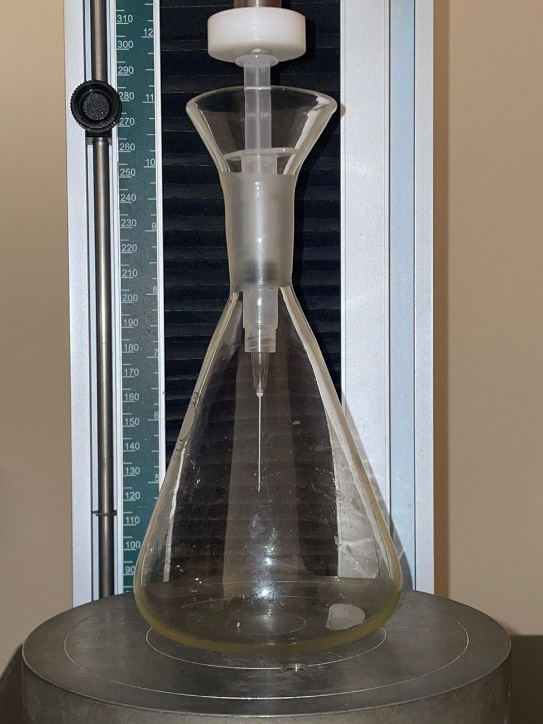


Supplementary Figure 1: injectability test of prepared hydrogel showing the setup of the 3 ml luer lock syringe with 22-gauge needle in the universal testing machine


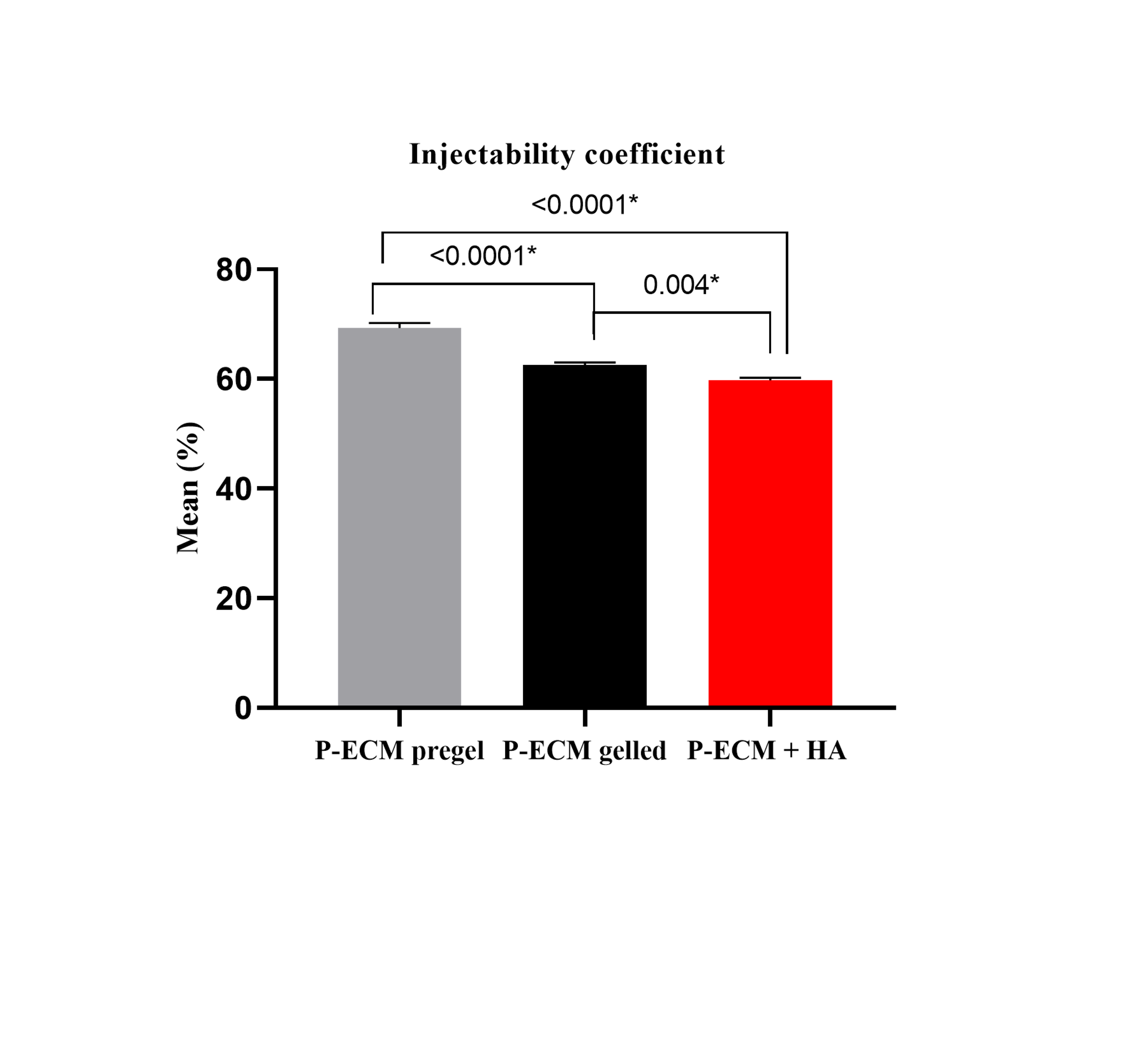


Supplementary figure 2: injectability coefficient of prepared hydrogels. P-ECM pregel had the highest injectability coefficient (69.27±0.94 %), followed by the gelled P-ECM (62.59 ±0.43 %) then the least injectability coefficient, was for the P-ECM + HA group (59.75±0.45 %). Significant differences were found between the groups.
